# Leveraging Ottawa Charter strategies to enhance COVID-19 vaccination: A systematic review of global health promotion approaches

**DOI:** 10.34172/hpp.025.43752

**Published:** 2025-05-06

**Authors:** Sara Pourrazavi, Behrouz Fathi, Zahra Fathifar, Haidar Nadrian, Hamid Allahverdipour

**Affiliations:** ^1^Research Center of Psychiatry and Behavioral Sciences, Tabriz University of Medical Sciences, Tabriz, Iran; ^2^Health Education & Promotion Department, Tabriz University of Medical Sciences, Tabriz, Iran; ^3^Department of Health Economics and Management, School of Public Health, Urmia University of Medical Sciences, Urmia, Iran; ^4^Department of Library, Tabriz University of Medical Sciences, Tabriz, Iran

**Keywords:** COVID-19, Health promotion, Systematic review, Vaccination

## Abstract

**Background:**

Despite global vaccination efforts, many countries struggled to achieve sufficient COVID-19 vaccination coverage. The use of Ottawa Charter health promotion strategies in vaccination programs not only enhances coverage but also fosters sustainable public health outcomes. This systematic review aims to identify actionable strategies to improve vaccination efforts.

**Methods:**

This systematic review involved a comprehensive literature search across PubMed, Scopus, Web of Science, and Embase, targeting studies published between January 2020 and August 2024. The search focused on government-led health promotion strategies for enhancing COVID-19 vaccination rates. Strategies were categorized five main areas of the Ottawa Charter for health promotion.

**Results:**

A total of 22 health promotion strategies were identified globally, categorized into five key areas based on the Ottawa Charter for health promotion. Notable strategies included engaging community, addressing misinformation, expanding vaccination sites, and providing culturally tailored communication.

**Conclusion:**

The findings underscore the significance of utilizing the Ottawa Charter framework to design inclusive and adaptable public health strategies that ensure equitable vaccination coverage globally.

## Introduction

 Vaccination has been one of the most effective tools for preventing infectious diseases, playing a crucial role in reducing the spread of COVID-19.^1,2^ Vaccinating a large portion of the population can theoretically reduce virus transmission; however, achieving herd immunity is influenced by factors such as emerging variants, waning immunity, and varying vaccine efficacy.^3^ Moreover, vaccination not only protects individuals but also safeguards vulnerable populations who cannot receive the vaccine, such as immunocompromised individuals.^3-5^ Therefore, vaccination is essential for reducing the burden on healthcare systems, saving lives, and enabling communities to return to normalcy.^3,6^

 Recognizing its importance, the World Health Organization set an ambitious global goal in 2021 to vaccinate 70% of the population to curb the COVID-19 pandemic.^7^ However, global vaccination efforts have faced challenges in achieving these targets due to several barriers. For example, misinformation, such as false claims about vaccine safety or efficacy, can erode public trust and create widespread hesitation.^8-10^ Safety concerns, fueled by both misinformation and rare adverse events, may lead to delays in vaccination decisions.^8-10^ Cultural resistance, on the other hand, reflects deeply rooted belief, traditions, or historical mistrust in healthcare systems, making it uniquely challenging to address with standardized messaging.^8-10^ Limited vaccine access, particularly in low-resource settings, further compounds these issues by creating physical and logistical barriers to achieving high coverage.^8-10^ These obstacles underscore the urgency of increasing vaccination coverage through multifaceted approaches that address vaccine hesitancy, distribution challenges, and misinformation.

 Many governments have adopted diverse strategies to promote health and increase vaccine uptake, including public education campaigns, improved access to vaccines, addressing hesitations, and encouraging public participation.^5,8,11,12^ While these initiatives are important, they alone are not sufficient to combat the multi-dimensional challenges presented by the pandemic. For instance, public education campaigns may lack sufficient reach in remote or underserved areas, and improved access does not fully address cultural resistance or deeply ingrained vaccine hesitancy. Additionally, these efforts often struggle to effectively combat the widespread misinformation that undermines trust in vaccination programs. Addressing these gaps requires more integrated and adaptable approaches to ensure a comprehensive response. According to existing evidence, actions that utilize a combination of health promotion strategies at different levels to prevent and combat a wide range of diseases and associated risk factors are more successful and cost-effective. For instance, integrated strategies that combine community education, policy advocacy, and enhanced healthcare accessibility have been shown to effectively reduce the prevalence of non-communicable diseases, as demonstrated in a study addressing cardiovascular health through community engagement and policy reforms.^13^ These findings highlight the value of comprehensive approaches in tackling current and future health challenges.

 The Ottawa Charter for health promotion, adopted in 1986 by the World Health Organization, offers a comprehensive framework for public health initiatives. Its five key areas—building healthy public policy, creating supportive environments, strengthening community action, developing personal skills, and reorienting health services—remain crucial, particularly in the context of a global pandemic, as they provide a structured approach to addressing health disparities, fostering community resilience, and ensuring equitable access to care during crises.^14,15^ Implementing these strategies can enhance public participation and satisfaction with vaccination programs, demonstrated by successful examples in countries like the UK.^16,17^ For instance, reorienting health services to include mobile vaccination units and prioritizing outreach in underserved communities have significantly improved access. Additionally, creating healthy environments through public education campaigns and transparent communication about vaccine safety has helped to build trust and counter misinformation.^8-10^ These measures have contributed to increased vaccination rates and more equitable healthcare policies, showcasing the impact of targeted interventions.

 While the Ottawa Charter remains an influential model for addressing global health inequalities, its implementation in the context of COVID-19 must be adaptable to local contexts. For instance, creating supportive environments for vaccination can be particularly challenging in regions with limited healthcare infrastructure, such as rural areas with sparse medical facilities or urban settings with overcrowded clinics.^18^ Highlighting these regional differences in infrastructure adds nuance to the adaptability point, emphasizing the need for context-specific strategies to effectively address barriers and enhance vaccination efforts.

 Therefore, applying the Ottawa Charter’s health promotion strategies to COVID-19 vaccination efforts can enhance vaccine coverage and promote long-term public health improvements. Tailoring these strategies to local conditions is crucial step in ensuring their effectiveness, as it highlights the importance of leveraging local knowledge to address specific regional barriers and resource limitations. These customized approaches can serve as valuable models for managing not only the current pandemic but also future public health crises. However, successful application of these strategies depends on a nuanced understanding of the unique challenges faced by each region.

 This study systematically evaluates global health promotion strategies for enhancing COVID-19 vaccination efforts, specifically focusing on their alignment with the Ottawa Charter’s five domains. We seek to answer the question: How have the five domains of the Ottawa Charter been applied in global health promotion strategies to enhance COVID-19 vaccination? By identifying actionable strategies and highlighting gaps in current approaches, this review aims to inform evidence-based interventions that address persistent challenges such as vaccine hesitancy, misinformation, and inequitable access. The findings are intended to guide future public health initiatives by providing practical recommendations for designing adaptable and culturally sensitive vaccination programs.

## Methods

 This systematic review aimed to identify and evaluate the health promotion strategies used by different countries to increase COVID-19 vaccination coverage, adhering to the Preferred Reporting Items for Systematic Reviews and Meta-Analyses (PRISMA) guidelines to ensure transparency and rigor.^19^

###  Search strategy and study selection 

 A comprehensive search was conducted across multiple databases, including PubMed, Scopus, Web of Science, and Embase, covering studies from January 2020 to August 2024. Search terms, structured using Boolean operators, included combinations of “COVID-19,” “vaccination,” “vaccine coverage,” “health promotion strategies,” “Ottawa Charter,” and “government.” The search was limited to English-language full-text articles. An example search strategy applied in PubMed database was as follows:

 (Community Actions[Title/Abstract]) OR (community mobilization[Title/Abstract]) OR (Community Participation[Title/Abstract]) OR (Social capital[Title/Abstract]) OR (Capacity Building[Title/Abstract]) OR (Community organization[Title/Abstract]) OR (Community engagement[Title/Abstract]) OR (Community involvement[Title/Abstract]) OR (Social empowerment[Title/Abstract]) OR (Ottawa charter[Title/Abstract]) OR (healthy Public Policy[Title/Abstract]) OR (Health Equity[Title/Abstract]) OR (Social determinant* of Health[Title/Abstract]) OR (Supportive Environment*[Title/Abstract]) OR (healthy environment*[Title/Abstract]) OR (Healthy setting*[Title/Abstract]) OR (Develop Personal Skill*[Title/Abstract]) OR (Health communication[Title/Abstract]) OR (Health literacy Reorient Health Service*[Title/Abstract]) OR (Health Care Reform[Title/Abstract]) OR (Inter sectoral Collaboration[Title/Abstract]) OR (Mediating[Title/Abstract]) OR (Enabling[Title/Abstract]) OR (Advocacy for health[Title/Abstract]) OR (healthy choice*[Title/Abstract]) OR (Non-governmental organization*[Title/Abstract]) OR (Media advocacy[Title/Abstract]) OR (Coalition[Title/Abstract]) OR (Empowerment[Title/Abstract]) OR (negotiating[Title/Abstract] OR Lobbying[Title/Abstract]) AND (“COVID-19 Vaccines”[MeSH Terms]) OR (“2019-nCoV Vaccine mRNA-1273”[MeSH Terms]) OR (“ChAdOx1 nCoV-19”[MeSH Terms]) OR (“Ad5-nCoV vaccine”[Supplementary Concept]) OR (“BIBP COVID-19 vaccine”[Supplementary Concept]) AND (“immunization coverage”[Title/Abstract]) OR (“uptake”[Title/Abstract]) OR (“distribution”[Title/Abstract]) OR (“product”[Title/Abstract]) OR (“policy”[Title/Abstract]) OR (“recommendation”[Title/Abstract]) OR (“delivery”[Title/Abstract]) OR (“Coverage”[Title/Abstract]) OR (“Covax”[Title/Abstract]) OR (“Coronavirus”[Title/Abstract]) OR (“COVID-19”[Title/Abstract]) OR (“COVID-19”[Title/Abstract]) OR (“SARS-CoV-2”[Title/Abstract]) OR (“Coronavirus”[MeSH Terms]) OR (“COVID-19”[MeSH Terms]) OR (“SARS-CoV-2”[MeSH Terms]) AND (“immunization coverage”[Title/Abstract]) OR (“vaccine uptake”[Title/Abstract]) OR (“vaccine distribution”[Title/Abstract]) OR (“vaccine product”[Title/Abstract]) OR (“vaccine policy”[Title/Abstract]) OR (“vaccine recommendation”[Title/Abstract]) OR (“vaccine delivery”[Title/Abstract]) OR (“Vaccination Coverage”[MeSH Terms]).

 After eliminating duplicates, two independent investigators (SP and ZF) reviewed abstracts, yielding 2654 articles. Moreover, we searched the reference lists of relevant articles by hand to identify further articles for analysis. Thereafter, eligible articles were selected for final analysis. The search strategy followed the guidelines of the Peer Review of Electronic Search Strategies statement.^20^

###  Inclusion and exclusion criteria

 The following criteria were established beforehand using the PICOS (population, intervention, comparison, outcomes, and time) design, and the research team (SP, ZF, HA) examined and approved the content validity:

*Population:* Studies that included population groups eligible to receive the COVID-19 vaccine.

*Intervention:* All interventions by the government to increase the coverage of COVID-19 vaccination and all evidences on measures to promote COVID-19 vaccination during the pandemic.

*Outcomes:* Studies that include information on vaccine prescribing, acceptance, and coverage.

*Time:* All English-language peer-reviewed journal articles published between January 2020 and August 2024 were included.

*Setting:* No limitations on the type of settings were imposed.

 Systematic reviews were excluded but were employed to identify additional eligible studies. Moreover, interventions that were not done by the government, protocols, conferential proceedings, articles not directly related to COVID-19 vaccination strategies, editorials, opinion pieces, and commentaries, and studies that were not open access and could not be obtained through interlibrary loans or authors who did not respond to requests were excluded. To ensure whether studies met the inclusion criteria, two authors (SP and ZF) conducted separate searches, screen the titles and abstracts, and then assessing the remaining 39 publications’ full texts.

###  Data extraction

**Figure 1:**
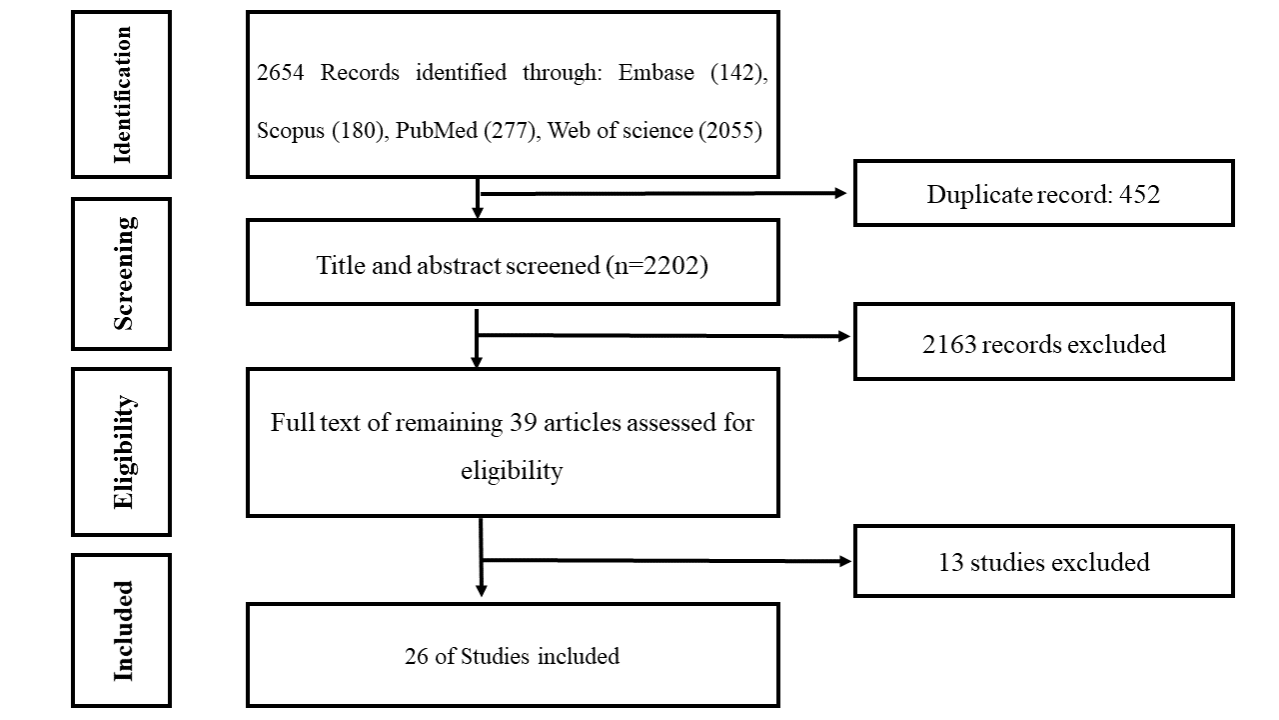
The diagram of the study based on PRISMA checklist

 Data were extracted from each selected study using a data extraction form which was developed by the research team. The extracted data included: first author and year of publication, country/region of study, description of vaccination strategy, outcomes measured (e.g., vaccination rates, public acceptance) and key findings and conclusions. The findings were synthesized using a qualitative narrative approach. Two members of the research team, SP and ZF, independently conducted a pilot test of the data extraction form using two out of the 39 selected articles. They compared their results, discussed discrepancies, and utilized the feedback to improve the form. The final version of the form was then used by SP to extract data from the remaining 37 articles, with ZF independently verifying the results. Both SP and ZF subsequently reviewed the full texts of the articles to cross-check their eligibility according to the predefined inclusion criteria and discrepancies were resolved through consensus under the supervision of a senior researcher (HA). As a result, 13 of the 39 articles were excluded, leaving a final sample of 26 studies (Figure 1).

###  Quality assessment

 The quality of the included studies was assessed using the Joanna Briggs Institute (JBI) Critical Appraisal Tools, appropriate for different types of studies (e.g., qualitative, cross-sectional). Each study was evaluated based on criteria such as clarity of research questions, methodological rigor, appropriateness of study design, data collection methods, and the validity and reliability of findings. Studies were classified as high, moderate, or low quality.

###  Data synthesis

 Data synthesis was conducted through a narrative approach. Key themes and strategies were identified and categorized based on the five main areas of the Ottawa Charter for health promotion:

1. Building healthy public policy
2. Creating supportive environments
3. Strengthening community action
4. Developing personal skills
5. Reorienting health services

## Results

Table 1 presents the main characteristics of the included studies (n = 26). Most studies report strategies implemented in the United States. The target populations include individuals eligible for vaccination, as well as low-income, underserved, and underrepresented communities, specific ethnic groups and minorities, school populations, healthcare workers, pharmacies, and public spaces.

**Table 1:** Health promotion strategies for increasing COVID-19 vaccination coverage

**Author(s)**	**Population & Country**	**Study Design**	**Strategies **	**Results **
Abdul-Mutakabbir et al, 2022^8^	Non-Hispanic Black and Hispanic/Latino individuals CA, USA		Engage community leaders Address misinformation Expand vaccination sites	Interventions improved vaccine willingness by up to 7% and increased uptake by 17% in some low-income and underserved communities.
Abdullah et al, 2022^11^	1760 Residents of five urban poor communities of Pakistan	Quasi-experimental study design	Providing accurate information Address misinformation Engage community leaders Mobile vaccination clinics	A collaboration between community organizations and academic institutions quickly boosted COVID-19 vaccine adoption among racially and ethnically marginalized groups.
Alcendor et al, 2022^12^	Minority communities in Middle Tennessee, USA	Descriptive case study	Mobile vaccination clinics	Vaccination rates increased among Black and non-Hispanic whites.
AuYoung et al, 2023^10^	California, USA	Descriptive study	Vaccine education and communication Engage community Address language and cultural barriers Utilizing technology Utilize local media	Ongoing vaccination services offered by pharmacies could play a significant role in boosting immunization rates, not only for COVID-19 but also for other diseases.
Baratta et al, 2023^1^	Italy		Prioritizing vulnerable populations Expand the health workforce	The Maximising Uptake Programme successfully vaccinated 7,979 individuals between February and August 2021 in the South West region of England, UK.
Berrou et al, 2022^5^	Ethnic minority groups and under-served communities in Bristol, North Somerset and South Gloucestershire, UK	Retrospective descriptive cohort study	Social marketing Address language and cultural barriers Engage community Use social media Address misinformation Provide incentives Expand vaccination sites Mobile vaccination clinics	
Bhattarai & Dhungana 2021^21^	Nepal		Securing vaccine supply agreements Donations and sharing of vaccine supplies Prioritizing vulnerable populations Ensuring equitable distribution of vaccines Expand vaccination sites	The targeted strategy effectively reduced disparities in vaccine coverage across different age groups and areas with varying COVID-19 risks. Over time, younger age groups experienced higher vaccination coverage, reflecting the age-based focus of the strategy.
Bhatti et al, 2022^22^	Canada	Observational, non-controlled implementation	Expand vaccination sites Implement mobile vaccination clinics Use social media and social marketing Address transportation barriers Provide accurate information and address misinformation Address language and cultural barriers Engage community leaders	By June 2022, vaccination coverage among all subgroups that had low rates in January 2022 showed a significant increase; however, Black students and those who speak multiple languages continued to have the lowest overall completion rates.
Carter et al, 2022^9^	Ontario, Canada.		Prioritizing vulnerable populations Expand vaccination sites Utilizing technology Address language and cultural barriers Engage community Implement mobile vaccination clinics Target underserved communities Provide accommodations for people with disabilities and underserved Collaboration and partnerships Vaccine education and communication	In the initial phase, the total number of individuals vaccinated was as follows: 24 224 in Province 1, 25 637 in Province 2,63,308 in Bagmati, 18 472 in Gandaki, 28 941 in Lumbini, 9420 in Karnali, and 14855 in Sudurpaschim.
Castillo et al, 2021^23^	Chile	Observational study	Strengthening logistics and supply chain Scaling up vaccine production Expand vaccination sites Securing vaccine supply agreements Utilizing technology	
Duchen et al, 2021^24^	Ontario, Canada		prioritizing vulnerable populations Utilizing technology	By October 19, 2021, approximately 88% of individuals aged 12 and older in the KFL&A region had received at least one dose of a COVID-19 vaccine, while nearly 85% had completed their vaccination with two doses.
Fairlie et al, 2023^2^	School Populations with Low Coverage — Seattle, Washington	Retrospective observational study	Address language and cultural barriers Engage community Targeted vaccination campaigns	
Günther et al, 2022^4^	England, Italy, and the USA		Prioritizing vulnerable populations Develop a prioritization framework	
Hamdan et al, 2023^6^	Malaysia		Prioritizing vulnerable populations Securing vaccine supply agreements	
Kaufman et al, 2022^25^	Australia	Mixed methods study	Community engagement	The results of this study suggest that the Vaccine Champions training program was largely effective, demonstrating significant impact and encouraging potential for diverse and resource-limited environments.
King et al, 2022^26^	Alberta, Canada		Use social media Prioritizing vulnerable populations Expand vaccination sites Utilizing technology Address language and cultural barriers Provide incentives Provide accurate information Engage community leaders	Over a period of four days, more than 1300 doses of the COVID-19 vaccine were administered to eligible Indigenous individuals.
Lin et al, 2021^27^	Taiwan	Observational, non-controlled program evaluation	Utilizing technology Expand the health workforce	As of October 28, 2021, Taiwan's first-dose COVID-19 vaccination rate surpassed 70%, and its full vaccination rate exceeded 30%, achieving these targets ahead of the end-of-month goal set by the Central Epidemic Command Center (CECC). The early accomplishment of these milestones marked significant progress in the country's vaccination campaign.
Marquez et al, 2021^28^	San Francisco, California	Mixed-method study	Engage community Provide incentives Provide accurate information and knowledge enhancing Train health workers Prioritizing vulnerable populations Social marketing Partnership with healthcare providers Expand vaccination sites Develop efficient vaccine delivery systems Address language and cultural barriers Expand the health workforce	Among the eligible individuals aged 16 and older living in the Mission District, 5.7% (n = 3590 out of 62 452) received at least one dose of the COVID-19 vaccine at the designated neighborhood vaccination site.
Mayfield et al, 2023^29^	Mecklenburg County, North Carolina, USA		Social marketing Engage community leaders Address language and cultural barriers Mobile health clinics	As of October 4, 2021, Mobile Health Clinics administered 15 459 first or single-dose COVID-19 vaccinations in Mecklenburg and nearby counties. The majority of those vaccinated through the MHCs were individuals from Black or Latinx communities, accounting for 63% of the total recipients.
Rojas-Ramirez et al, 2022^30^	Kentucky, UK		Expand the health workforce Train health workers Expand vaccination sites Utilizing technology	This effort began in January 2021, operating six days a week, and provided over 2000 COVID-19 vaccines each day.
Selembo et al, 2022^31^	New Hampshire, US		Prioritizing Vulnerable Populations Engage Community	
Swope et al, 2023^32^	Southern California	Quality-improvement implementation study	Utilizing technology Mobile vaccination clinics	By the end of 2021, Kaiser Permanente Southern California successfully achieved a 70% vaccination rate across 83% of the 670 ZIP codes it serves, leading to an overall vaccination rate of 81% for that year. Additionally, more than two-thirds of those vaccinated through the targeted mobile health units were either Hispanic or Black.
Waitzberg et al, 2021^33^	Israel	Qualitative, descriptive analysis	Implement information systems	The GPP served as a flexible public health tool, adaptable based on changing epidemiological needs. The article emphasizes important considerations for policymakers when formulating vaccine certificate policies, including ensuring fairness, phased implementation, and robust enforcement mechanisms.
Wallia et al, 2021^34^	Health care workers in Illinois	Case study	Use social media	
Warren & Lofsted, 2022^35^	France, Germany, Sweden, Switzerland and England within the UK		Implement health information systems (France, Switzerland, UK) Partnership with healthcare providers and expand workforce (Germany) Engage community (Germany, UK and Sweden) Mobile vaccination Clinics (Sweden, UK) Targeted vaccination Campaigns (Switzerland) Expand vaccination sites (UK) Prioritizing vulnerable populations (France, Germany, Sweden, Switzerland, UK)	In the short-term, France's Pass Sanitaire policy spurred a rapid increase in vaccinations, with Prime Minister Jean Castex reporting that over 790 000 individuals were vaccinated within 24 hours on July 13. This surge in vaccinations resulted in a roughly 15% increase in first-dose uptake over the previous month, following a period of stagnation in vaccination rates.
Wu et al, 2022^36^	Michigan	Needs assessment survey	Engage community Expand vaccination sites Mobile vaccination clinics Utilizing technology	Despite facing obstacles, mobile clinics utilizing GIS mapping successfully expanded access to COVID-19 vaccines. Community-driven partnerships across multiple sectors played a key role in boosting capacity and ensuring that hard-to-reach populations were able to receive vaccinations. To date, the Eastern Michigan University CHDIS mobile clinics have reached over 3700 individuals in underserved areas of Michigan, helping to bridge gaps in vaccine accessibility.

###  Risk of bias

 The critical appraisal of the studies using the JBI Critical Appraisal Tools reveals that 37.04% of the studies are of high quality, 62.96% are of moderate quality, and none are of low quality (Table 2).

**Table 2:** Quality Assessment of Studies Using JBI Critical Appraisal Tools

**Quality**	**Studies**
High Quality (75-100%)	Abdul-Mutakabbir et al,^8^ AuYoung et al,^10^ Baratta et al,^1^ Bhatti et al,^22^ Castillo et al,^23^ Kaufman et al,^25^ Mayfield et al,^29^ Rojas-Ramirez et al,^30^ Waitzberg et al,^33^ Wu et al^36^
Moderate Quality (50-74%)	Abdullah et al,^11^ Alcendor et al,^12^ Bhattarai & Dhungana,^21^ Berrou et al,^5^ Carter et al,^9^ Duchen et al,^24^ Fairlie,^2^ Günther et al,^4^ Hamdan et al,^6^ King et al,^26^ Lin et al,^27^ Marquez et al,^28^ Selembo et al,^31^ Swope et al,^32^ Wallia et al,^34^ Warren & Lofstedt^35^
Low Quality (0-49%)	-

## Health promotion strategies

 Twenty-two health promotion strategies aimed at increasing COVID-19 vaccination coverage are identified globally. These strategies are categorized into the five areas outlined by the Ottawa Charter for health promotion.

###  Building public policies

 One of the key principles of the Ottawa Charter is the creation of public policies that promote health. These policies can include laws and regulations that facilitate access to vaccines and reduce economic, social, and geographical barriers.

####  Securing vaccine supply agreements

 Governments and international organizations secure vaccine supply agreements with vaccine manufacturers to ensure their populations have access to vaccines. These agreements include both purchasing and distribution arrangements.

 In December 2020, Nepal amended its Drugs Act 2035 to allow emergency registration of new drugs and vaccines, enabling manufacturers to import products not yet listed by the WHO. On January 13, 2021, Nepal’s Department of Drug Administration (DDA) invited global vaccine manufacturers to apply for product registration. The Serum Institute of India quickly applied for its Covishield vaccine, developed by the Oxford-AstraZeneca, and by January 15, 2021, the DDA had registered Covishield for emergency use in Nepal.^21^

####  Donations and Sharing of Vaccine Supplies

 Some countries with excess vaccine supplies donated or shared doses with countries that had limited access. This included donations from high-income countries to low- and middle-income countries through international organizations such as COVAX.

 After the visit of Nepal’s Minister for Foreign Affairs to India, the country provided Nepal with 1 million doses of the Covishield vaccine. Additionally, China pledged 500 000 doses of its COVID-19 vaccine to Nepal. These vaccine donations were used as diplomatic tools to help Nepal and other nations accelerate the resumption of tourism and trade.^21^

####  Implement information systems

 Implementing information systems can improve vaccine management and monitoring, such as issuing vaccine cards and passes for public places.

 On July 12, President Macron announced that starting in August, a “Pass Sanitaire” would be required to access cafes, restaurants, shopping centers, long-distance public transport, and medical establishments in France. In Switzerland, a COVID-19 vaccine certificate was introduced in June 2021, but its initial enforcement by businesses was weak, which lead to disappointment from the Health Minister. However, starting September 13, the Swiss Federal Council made the certificate mandatory in restaurants, leisure, and cultural venues until January 2022, primarily to reduce hospitalizations rather than solely to increase vaccine uptake. Additionally, there are plans to introduce a vaccine or immunity passport for entry into nightclubs and large indoor venues, though pubs and bars will be exempt.^35^

####  Collaboration and partnerships

 Collaboration and partnerships between governments, international organizations, and other stakeholders help address health infrastructure challenges. This includes sharing expertise, knowledge, and resources to strengthen health infrastructure.

 In mid-March, Germany allowed general practitioners and family doctors to administer COVID-19 vaccines. This revitalize the vaccination campaign, as these healthcare providers are widely trusted and accessible across the country. Their involvement significantly boosted vaccination rates and increased acceptance, particularly among hesitant groups.^35^

###  Creating supportive environments

 Creating supportive environments involves establishing conditions where individuals can access vaccines easily and with confidence. This includes setting up vaccination centers in accessible locations such as schools, mosques, shopping centers, and deploying mobile vaccination units to reach remote and underserved areas.

####  Social marketing and utilizing media

 Social marketing campaigns play a crucial role in increasing vaccine acceptance by addressing hesitancy and highlighting the benefits of vaccination. These campaigns can be delivered through television, radio, billboards, social media, and other communication channels.

 In the UK, regions like Bristol, North Somerset, and South Gloucestershire placed pull-up banners and posters in all vaccination clinics, encouraging people to share their vaccination experiences with family and friends.^5^ In California, community outreach efforts included distributing flyers and posters, while the Unidos en Salud (“United in Health”, UeS) initiative conducted Spanish-language radio, newspaper, and television interviews to promote their neighborhood vaccination site. Community leaders also shared their vaccination experiences on social media platforms like Facebook and TikTok to inspire others to get vaccinated.^28^

 Additionally, to reach farmworkers without internet access, UC Davis Workgroup members collaborated with Radio Bilingüe, a national Latino public radio network. They implemented a strategic communication framework that focused on persuasive communication rather than merely providing information. This included creating culturally appropriate social media-ready short films and videos featuring trusted messengers, with content delivered in the necessary languages.^10^

####  Address transportation barriers

 Improving access to vaccines requires addressing transportation barriers, including offering transportation to vaccination sites and setting up vaccination centers in areas accessible by public transportation. In Canada, practical measures such as arranging transportation and extending clinic hours helped clients who faced challenges attending appointments due to work or transportation issues.^22^

####  Expand vaccination sites

 Expanding the number of vaccination sites can significantly enhance vaccine accessibility and distribution. Governments established vaccination centers in various locations, including community centers, pharmacies, workplaces, hotels, mosques, churches, supermarkets, parks, community halls, fire stations, doctor’s offices, school parking lots, and other familiar public spaces.^5,9,22,26,28,35,36^

####  Address language and cultural barriers

 Promoting COVID-19 vaccination coverage requires addressing language and cultural barriers. This involves providing materials in multiple languages and using culturally appropriate communication strategies.

 Berrou et al outlined several strategies used in the UK to increase COVID-19 vaccine uptake by addressing language barriers and community concerns, including:

- Small-scale webinars conducted in community languages, such as within the Somali community and House of Praise, which helped alleviate concerns and encourage vaccine sign-ups.
- Revising COVID-19 vaccine appointment text message invitations with more positive language, translated into eight languages.
- Developing a Language Hub on the Healthier Together website, offering trusted resources in 22 different languages.
- Creating and distributing community-led videos and leaflets in multiple languages, often produced by organizations embedded in local communities.^5^

In Seattle, school-based vaccination clinics implemented strategies to overcome cultural and linguistic barriers. Seattle Public Schools maintained weekly communication with families via email, phone calls with prerecorded messages, and text messages through the Talking Points platform, which supported six languages. The district also distributed multilingual communication toolkits developed by Public Health—Seattle & King County to parent-teacher-student associations and community organizations. Vaccine providers were selected for their cultural competency, such as an independent Black-owned pharmacy with staff fluent in several African languages.^36^

####  Target underserved communities

 Focusing on underserved communities can reduce disparities in vaccine access and increase uptake. This includes establishing vaccination sites in underserved areas and deploying mobile vaccination clinics. In the UK, efforts targeted individuals experiencing homelessness, non-English speakers, minority ethnic groups, refugees, asylum seekers, gypsy, Roma, and traveler communities, boat dwellers, and people with limited access to vaccination centers. This also included individuals with learning disabilities, serious mental illness, substance dependencies, physical and sensory impairments, and dementia.^5^

####  Provide incentives

 Offering incentives, such as free transportation or gift cards, can encourage vaccine uptake, particularly among populations facing barriers, such as low-income individuals or those in remote areas. Examples include sharing traditional foods at Métis gatherings in Alberta, subsidized food programs in Ontario and the UK, and free walk-up testing in San Francisco for Latinx and low-income community members, along with support for those who tested positive.^5,22,26,28^

###  Strengthening community action for health

 The Ottawa Charter highlights the importance of community participation in health decision-making. Involving local leaders, religious groups, NGOs, and other social entities can bolster health initiatives.

####  Community engagement

 Engaging with communities is essential for building trust and increasing vaccination coverage. Involving community and religious leaders can help address concerns, combat misinformation, and improve vaccine acceptance. In Ontario, strategies included the involvement of Indigenous Elders, collaboration with community partners (e.g., primary care champions), hosting community events, and leveraging preexisting relationships.^22^

 In California, public health efforts were strengthened by pre-existing relationships from earlier collaborations. Community groups, ethnic media, schools, and faith-based organizations engaged virtually to answer questions and address information needs. Trusted community messengers, including health workers and faith leaders, were paired with scientific experts to build credibility and trust. These strategies emphasized trust, cultural relevance, and community engagement as key components of public health communication.^10^

 In Germany, the UK, and Sweden, celebrities, key politicians, and medical advisors were involved to promote vaccine campaigns.^35^

####  Targeted vaccination campaigns

 Targeted vaccination campaigns can help address distribution challenges in specific populations, including migrant workers, homeless individuals, and refugees. In Switzerland, the Federal Office of Public Health launched a solidarity campaign that emphasized the altruistic nature of vaccination, highlighting that it protects both individuals and the wider community.^35^

###  Developing personal skills

 Developing personal skills involves educating and informing people about the importance of vaccination and how to protect themselves and others against COVID-19. This can be done through educational programs, brochures, instructional videos, enhancing health literacy, and individual counseling.

####  Peer influence

 Peer influence is a powerful tool in increasing vaccine acceptance. Encouraging vaccinated individuals to share their experiences with family and friends can reduce vaccine hesitancy and increase vaccination coverage. Marquez et al describe an initiative where vaccinated individuals were empowered as “vaccine ambassadors” to influence others. Staff encouraged clients to share positive vaccination experiences with unvaccinated friends, family, and co-workers. These ambassadors dispelled myths, modeled good health behavior, and provided social support for vaccination. They were given flyers with site information and a referral phone number for vaccine-related questions.^28^

####  Provide accurate information and knowledge enhancing

 Providing accurate information about the COVID-19 vaccine can address concerns and reduce vaccine hesitancy. This includes sharing details about the vaccine’s safety, efficacy, development, and testing processes. In San Francisco, California, the health team employed various methods to inform community members about vaccine benefits and eligibility, boosting demand for vaccination.^28^

####  Vaccine education and communication

 Educating the public about vaccine safety, efficacy, and benefits can help address hesitancy and increase uptake. In California, a working group focused on community engagement, social justice, and cultural humility. The group, which included academic and community partners, initially concentrated on safety guidelines and vaccine trials, later hosting town halls and virtual sessions to address concerns about vaccine safety. They also used in-person outreach at health fairs and distributed free PPE kits alongside vaccine information to promote overall safety. In San Diego, art-based approaches such as murals and exhibitions were employed to communicate COVID-19 messaging and foster community healing.^10^

###  Reorienting health services

 Reorienting health services involves improving the quality and accessibility of healthcare. In the context of COVID-19 vaccination, this includes training and equipping healthcare workers, enhancing the vaccine supply chain, and using modern technologies for vaccination registration and follow-up.

####  Strengthening logistics and supply chain

 Ensuring a strong and efficient vaccine supply chain is essential to overcoming distribution challenges. This involves improving transportation infrastructure, increasing storage capacity, and enhancing distribution networks. Castillo et al highlighted that Chile’s existing infrastructure, developed after the H1N1 flu pandemic, played a significant role in the success of its COVID-19 vaccination campaign. The National Network of Vaccine and Immunoglobulin Deposits, with 26 centers, helped address logistical challenges, including the need for ultra-cold storage for the Pfizer-BioNTech vaccine. This infrastructure, combined with the cold chain systems already in place at all primary healthcare centers through the National Immunization Plan, ensured broad vaccine access.^23^

####  Prioritizing equity

 vulnerable populations such as the elderly, healthcare workers, and those with underlying health conditions. Ontario’s three-phase vaccination strategy, released in December 2020, prioritized specific groups:

*Phase 1:* Focused on seniors in congregate living settings, healthcare workers, Indigenous populations, chronic homecare recipients, and adults aged 80 and older.

*Phase 2:* Expanded to adults aged 60–79 (in five-year increments), individuals in high-risk congregate settings (e.g., shelters, penitentiaries), people with chronic health conditions, caregivers, and those unable to work from home.^24^

 In Nepal, the Ministry of Health and Population prioritized frontline workers, including health and sanitation personnel, ambulance and hearse drivers, for the first phase. The second phase extended to remaining frontline workers, security personnel, elderly individuals in shelters, inmates, health professionals, journalists, diplomats, and government administrators.^21^

####  Utilizing technology

 Technology can help address distribution challenges by providing real-time data, improving vaccine tracking, and enhancing supply management. In Ontario, Canada, a government-sponsored research institute consolidated data from multiple sources, sharing it with scientists and public health authorities to create a more efficient and equitable vaccination strategy. Real-time vaccine coverage data was used by the government, health units, and community organizations to make informed decisions.^24^

 Carter et al described the use of ArcGIS Pro and ArcGIS Online software to map vaccination coverage in Kingston, Frontenac, Lennox, and Addington. The data were used to measure health disparities in vaccination coverage by factors like material deprivation, sex, and urban/rural status. These insights allowed for targeted interventions in the most marginalized groups.^9^

 GIS technology was also utilized by the EMU CHDIS team in Michigan to optimize mobile vaccination clinics for underserved African American communities. Spatial analysis helped identify priority locations, such as community centers, places of worship, and federally qualified health centers. This strategic planning improved vaccine distribution to priority populations.^36^

####  Mobile vaccination clinics

 Mobile vaccination clinics are essential for improving access to vaccines in remote and underserved areas. This includes deploying mobile clinics in rural regions and offering in-home vaccinations for individuals who are homebound. Southern California, for instance, redirected mobile health vehicles to underserved areas to increase COVID-19 vaccination coverage.^32^

 In the Stockholm region, there was a significant disparity in vaccination rates between wealthier and poorer areas. To address this, local health officials introduced vaccination buses and pop-up tents, particularly targeting foreign-born individuals. In the UK, pop-up vaccination centers were even set up at nightclubs as part of a health promotion strategy.^35^

####  Develop efficient vaccine delivery systems

 Efficient vaccine delivery systems ensure that vaccines reach their target populations in a timely and organized manner. This includes implementing clear vaccination schedules and increasing the capacity for vaccine appointments. For example, clinics in San Francisco increased their appointment capacity to 500 per day to improve vaccination coverage.^28^

####  Expand the health workforce

 Expanding the health workforce helps increase vaccine delivery capacity, allowing more people to be vaccinated. This involves recruiting and training additional health workers, including nurses, pharmacists, and community health workers. In Taiwan, pharmacies were used to provide vaccination services. In the UK, dentists were allowed to administer COVID-19 vaccines, and in California, the number of staff at vaccination sites was increased.^27,28,30^

####  Train health workers

 Health workers need thorough training on the importance of vaccination, vaccine safety, and proper administration techniques. Ongoing training and support ensure they have the knowledge and skills to deliver vaccines effectively. In Kentucky, health worker training was divided into four sections: general vaccination concepts, COVID-19-specific information, proper muscle injection techniques, and practical training. The University of Kentucky developed an online course using Canvas® software for all College of Dentistry providers, including faculty, assistants, and students. The training was overseen by nursing faculty, and dental faculty members were required to complete CPR training for license accreditation.^30^

## Discussion

 This study aims to identify actionable health promotion strategies for countries to improve vaccination rates and more effectively combat the COVID-19 pandemic. A total of 22 health promotion strategies were identified. These strategies, categorized under the five Ottawa Charter domains, offer a comprehensive understanding of the multifaceted approaches required for successful vaccine distribution. Each strategy underscores the importance of coordinated efforts across public policy, supportive environments, community engagement, personal skill development, and reorientation of health services.

 Building public policies has played a crucial role not only in securing vaccine supply agreements and donations but also in implementing vaccine passports and shaping global health governance during the pandemic.^6,21,23,33,35^ Governments worldwide enacted legal and regulatory frameworks essential for the rapid procurement and distribution of vaccines. For example, Nepal introduced legislative changes to expedite vaccine imports, demonstrating how regulatory flexibility became a vital tool in crisis management. Similarly, countries like India and China engaged in “vaccine diplomacy,” using vaccines to strengthen bilateral relations and promote cooperation with neighboring countries such as Nepal. These collaborations helped facilitate the cross-border flow of vaccines, contributing to regional stability in vaccine availability.^21^

 Vaccine passports emerged as a critical policy tool, balancing public health protection with the reopening of economies. France’s “Pass Sanitaire” and Switzerland’s COVID-19 certificate system exemplify how these passports were used to regulate access to public spaces, travel, and large events. Although effective in enforcing public health measures, the implementation of these passports varied significantly based on local contexts and public acceptance. In countries with high trust in government and strong public health infrastructure, vaccine passports were widely embraced. However, in regions with greater skepticism toward vaccination or government policies, these measures faced resistance,^35^ emphasizing the importance of cultural and societal factors in public policy enforcement.

 Trust in national public health authorities plays a crucial role in influencing vaccine acceptance, even in countries where public health is primarily managed at subnational levels.^37^ Government policies, including non-pharmaceutical interventions, indirectly affected vaccine acceptance, with stringent measures correlating positively with acceptance, especially in settings with weaker social norms and lower trust.^38^ However, vaccine mandates encountered resistance in some regions due to cultural beliefs, misinformation, and skepticism.^39^ These findings underscore the complex interplay between trust, social norms, and government policies in public health, underscoring the need for tailored, context-specific vaccination strategies.

 Moreover, these vaccine-related policies were not just a short-term response to the pandemic but also helped prepare for future global health crises. By fostering international partnerships and developing flexible legal frameworks, governments demonstrated that cooperation, adaptability, and innovation are essential in tackling complex health challenges.^35^ Public policies, therefore, served not only as a tool for immediate crisis management but also as a means to strengthen global health systems for the long term.

 Creating supportive environments through social marketing and media utilization proved vital in promoting vaccine uptake. Community-specific approaches, such as using culturally tailored media and multilingual communication strategies, were particularly effective in engaging hard-to-reach populations, such as migrant workers and non-English speakers.^10,28^ Addressing logistical barriers, like transportation and expanding vaccination sites, also improved access in underserved communities.^22^ The culturally competent outreach seen in the UK and Seattle illustrates how targeted communication can overcome language and cultural barriers, boosting overall vaccination coverage.^2,5^

 Social media platforms have shown great potential in enhancing vaccination uptake and coverage. Studies indicate that Twitter and Facebook users who rely on these platforms for health information are more likely to be vaccinated.^40^ Social media can effectively deliver vaccination messages in local languages and dialects, making the information more comprehensible and acceptable to target audiences.^41^ In linguistically diverse communities, providing information in the native language improves access to vital data and alleviates concerns. Additionally, trusted community figures and local leaders often hold significant influence in collective decision-making. Leveraging social media to share supportive messages through these figures can directly impact vaccine acceptance within communities.

 Additionally, in some societies, specific symbols and values are prioritized. Leveraging these cultural symbols in vaccination campaigns on social media can enhance communication and make messages more relatable and engaging for the target audience.^42^ For instance, in cultures that place a central value on family, emphasizing family protection through vaccination can be particularly impactful.^43,44^ Furthermore, vaccination messages rooted in culturally relevant stories and experiences can create deeper emotional connections, fostering empathy and increasing individuals’ willingness to get vaccinated.^44^

 In strengthening community action for health, engaging with local leaders, religious figures, and influential community members was crucial for building trust and improving vaccination efforts. Examples from California, Germany, and Sweden illustrate how mobilizing these trusted individuals helped address vaccine hesitancy and counter misinformation within communities. By leveraging pre-existing relationships, these efforts promoted dialogue and education, ensuring that people received accurate and trusted information.^10,28,35^

 Collaboration with community organizations and faith leaders helped reach marginalized populations, ensuring that vaccine information was delivered in a culturally sensitive manner.^10^ Switzerland’s solidarity campaign further illustrated how framing vaccination as both an individual and collective responsibility fostered a sense of communal duty. By emphasizing the protection of vulnerable groups, the campaign motivated individuals to participate in the broader public health effort, benefiting both personal health and societal well-being.^35^ This sense of shared responsibility and mutual care effectively increased vaccination rates and mitigated hesitancy. Through these initiatives, it became evident that community engagement and cultural competence are essential for building resilient public health systems and encouraging widespread participation in health-promoting behaviors, especially during crises.

 Developing personal skills was significantly bolstered by peer influence and targeted education campaigns. Empowering vaccinated individuals as “vaccine ambassadors” proved a powerful strategy for fostering vaccine acceptance, as these ambassadors shared personal experiences within their social networks. The use of personal stories added a relatable and trusted dimension to vaccine promotion, allowing people to hear directly from those they know, bridging gaps in understanding and trust. This method not only enhanced the reach of official health messaging but also increased its impact, as it came from familiar sources.^28^

 Moreover, equipping individuals with clear, accessible, and accurate information about vaccine safety, efficacy, and the development process helps dispel fears and uncertainties. Outreach programs should emphasize transparency, addressing common concerns like potential side effects and debunking myths about vaccines. Transparent communication about vaccines, including potential negative aspects, is crucial for maintaining public trust and combating misinformation. While disclosing negative information may temporarily decrease vaccine acceptance, it ultimately increases trust in health authorities and reduces the spread of conspiracy theories.^45^ The unprecedented speed of COVID-19 vaccine development necessitates even greater transparency to maintain public trust.^46^ Clear and timely communications about adverse events during vaccine trials is essential to prevent confusion and misinformation.^46^ Overall, these studies emphasize the importance of transparent, fact-based vaccine communication to support informed decision-making and maintain long-term trust in health authorities.

 Finally, reorienting health services played a crucial role in ensuring that the vaccine delivery system was both robust and adaptable to changing circumstances. Countries like Chile leveraged their well-established cold chain infrastructure to maintain vaccine efficacy across diverse regions, which was key to ensuring equitable vaccine distribution, even in remote areas.^23^ Similarly, mobile clinics in Stockholm and Southern California were instrumental in reaching underserved populations, particularly those living in geographically isolated or socioeconomically disadvantaged regions.^32,35^ These mobile clinics served as both vaccination sites and educational hubs, helping to bridge the gap in healthcare access.

 Expanding the health workforce was also essential to meet the growing demand for vaccine administration. By training additional healthcare personnel and incorporating non-traditional vaccine administrators, such as pharmacists and dentists, vaccination campaigns gained scalability and flexibility. This approach not only alleviated pressure on overburdened healthcare systems but also increased vaccine accessibility for a broader portion of the population.^1,27,28,30^ The use of non-traditional administrators proved particularly valuable in areas with healthcare personnel shortages, where local pharmacies and dental clinics served as convenient vaccination sites, reducing logistical barriers for individuals in need of vaccination.^1,27,28,30^

 The integration of technology into the reorientation of health services—through digital health records, appointment scheduling, and tracking vaccine stock—enabled health systems to streamline processes and efficiently monitor progress. Digital tools offer significant potential to enhance immunization programs and improve vaccine distribution. For example, block chain technology can improve vaccine stock management, logistics, and transparency in distribution.^47^ These tools can help address barriers to immunization, enhance vaccine coverage, and support the surveillance of vaccine-preventable diseases and adverse events.^48^ Overall, digital transformation in immunization services improves data management, supply chain logistics, and healthcare infrastructure efficiency.

 These combined efforts to reorient health services ensured that vaccination programs were responsive to immediate needs and prepared to adapt to future public health challenges.

## Limitations

 Potential limitations of this systematic review include selection bias, publication bias and language bias (limited to open access and English publications). This could impact the comprehensiveness of the findings, as some valuable data from non-open access or non-English studies may have been omitted. Additionally, rapid changes in COVID-19 vaccination policies and strategies might lead to the inclusion of studies that may quickly become outdated.

## Conclusion

 The global strategies for promoting COVID-19 vaccination highlight the critical role of the Ottawa Charter’s health promotion framework in guiding multi-sectoral collaboration and innovative public health interventions. By addressing challenges like vaccine hesitancy and access disparities through culturally tailored outreach, community engagement, and logistical improvements, these efforts demonstrate the value of adaptable and equitable approaches in increasing vaccination coverage and safeguarding global communities.

## Implications for practice and future research

 Health promotion strategies should prioritize underserved communities, including ethnic minorities, low-income populations, and those in remote areas, ensuring equitable access to vaccination services. This includes establishing vaccination sites in familiar and accessible locations, such as community centers, schools, and places of worship. Governments, international organizations, and healthcare providers should enhance collaboration to improve logistical infrastructure, including vaccine supply and distribution, particularly in low- and middle-income countries.

 Public health campaigns should utilize culturally appropriate messaging and multiple languages to address vaccine hesitancy, especially in communities with language barriers. Engaging community leaders and leveraging social marketing can help build trust and enhance vaccine acceptance.

 Future studies should assess the long-term impact of culturally and linguistically tailored outreach strategies on improving vaccine uptake across diverse populations. Research should also examine the sustainability and adaptability of these strategies for future pandemics or vaccination efforts beyond COVID-19. Comparative research across countries is needed to evaluate the scalability of successful vaccination strategies, particularly those involving partnerships, mobile clinics, and community engagement. Additionally, further research should explore the specific barriers faced by marginalized groups, such as individuals with disabilities or chronic illnesses, and how tailored interventions can bridge these gaps.

## Competing Interests

 Hamid Allahverdipour is Editor-in-Chief of the Health Promotion Perspectives. Haidar Nadrian is the Associate Editor of the Health Promotion Perspectives. Other authors declare no competing interests.

## Ethical Approval

 This research was performed based on Tabriz University of Medical Sciences ethics committee approval (Approval ID: IR.TBZMED.REC.1401.652).
